# Carbohydrate Diet and Reproductive Performance of a Fruit Fly Parasitoid, *Diachasmimorpha tryoni*

**DOI:** 10.1673/031.013.7401

**Published:** 2013-07-30

**Authors:** Ashley Louisa Zamek, Olivia Louise Reynolds, Sarah Mansfield, Jessica Louise Micallef, Geoff Michael Gurr

**Affiliations:** 1 Cooperative Research Centre for National Plant Biosecurity, University of Canberra, Bruce ACT 2601, Australia; 2 EH Graham Centre for Agricultural Innovation, NSW Department of Primary Industries and Charles Sturt University, Elizabeth Macarthur Agricultural Institute, Private Bag 4008, Narellan, NSW 2567, Australia; 3 Faculty of Agriculture and Environment, University of Sydney, Australian Technology Park, Eveleigh, NSW 2015, Australia; 4 EH Graham Centre for Agricultural Innovation, NSW Department of Primary Industries and Charles Sturt University, PO Box 883, Orange, NSW 2800, Australia; 5 Current address: School of Environmental and Rural Science, University of New England, Armidale NSW 2351 Australia

**Keywords:** biological control, honey, ovigeny, sugar

## Abstract

Augmentative releases of parasitoid wasps are often used successfully for biological control of fruit flies in programs worldwide. The development of cheaper and more effective augmentative releases of the parasitoid wasp *Diachasmimorpha tryoni* (Cameron) (Hymenoptera: Braconidae) may allow its use to be expanded to cover Queensland fruit fly, *Bactrocera tryoni* (Froggatt) (Diptera: Tephritidae), a serious pest of many vegetables and most fruit production in Australia. This demands a fuller understanding of the parasitoid's reproductive biology. In this study, mating status, fecundity, and size of female *D. tryoni* were determined under laboratory conditions. A range of pre-release diets, 10% concentrations of honey, white sugar, and golden syrup, were also assessed in the laboratory. Mature egg loads and progeny yields of mated and unmated parasitoid females were statistically similar, demonstrating that mating status was not a determinant of parasitoid performance. Female lifespan was not negatively impacted by the act of oviposition, though larger females carried more eggs than smaller individuals, indicating a need to produce large females in mass-rearing facilities to maintain this trait. White sugar gave the highest adult female lifespan, while honey and golden syrup shared similar survivorship curves, all significantly greater compared with water control females. Pre-release feeding of *D. tryoni*, particularly with white sugar, may enhance the impact of released parasitoids on *B. tryoni*. These findings are important because honey is currently the standard diet for mass-reared braconids, but white sugar is less than one-third the cost of other foods; however further work is required to assess postrelease performance of the parasitoid.

## Introduction

The Queensland fruit fly, *Bactrocera tryoni* (Froggatt) (Diptera: Tephritidae), is the major fruit fly pest of eastern Australia, with literature on its impact and control dating back more than 115 years (Clarke et al. 2011). Decreasing availability of allowable insecticides has led to the need to explore additional tactics incorporating biological control in an integrated pest management system for *B. tryoni*. The parasitoid *Diachasmimorpha tryoni* (Cameron) (Hymenoptera: Braconidae) is endemic to Australia and was successfully introduced into Hawaii in 1913 (Duan and Messing 1999). *D. tryoni* was later used successfully on Maui, Hawaii, in an augmentative release (Wong et al. 1991) and subsequently in a concurrent parasitoid and sterile fly release program (Wong et al. 1992) to suppress a wild population of the Mediterranean fruit fly, *Ceratitis capitata* (Wiedemann). It is also used effectively along the Mexican/Guatemalan border (Sivinski et al. 2000) for the control of *C. capitata*.

Lack of suitable sugar sources for adult parasitoid wasps is recognised as an important cause of failure in biological control programs (McDougall and Mills 1997; Bautista et al. 2001). Sugar (carbohydrate) consumption is known to increase the lifespan and fecundity of many parasitoid species (Siekmann et al. 2001), which is why many mass-rearing facilities rear adults on honey or honey solutions (Cancino and Montoya 2006). Many parasitoids, including *D. tryoni*, require carbohydrates as a source of energy (Jervis et al. 1993; Wäckers 2001), which are provided by the consumption of sugar-rich foods (Wyckhuys et al. 2008). The carbohydrate food sources provided to parasitoids are important for increasing adult lifespan, which directly influences the effectiveness of these parasitoids in biological control programs (Jacob and Evans 2000).

The effect of different food sources on lifespan and fecundity have not been explored for *D. tryoni*; however, honey is known to increase lifespan in other braconids, including *Fopius arisanus* (Sonan) (Wu et al. 2008), *D. longicaudata* (Ashmead) (Sivinski et al. 2006), and *D. kraussii* (Fullaway) (Duan 2000). However, it is important to gain species-specific information, especially in terms of food source, in order to support mass-rearing systems and optimise biological control outcomes. Another potentially important factor that has not been investigated for *D. tryoni* is the effect of oviposition on female lifespan. Research indicates that there is an energetic cost to the act of reproduction (Wu et al. 2008), which may shorten the lifespan of female *D. tryoni*.

Similarly, knowledge of the reproductive biology and ecology of parasitoids is crucial when developing biological control programs based on augmentative releases (Eliopoulos et al. 2003). *D. tryoni* is a synovigenic species(Ramadan et al. 2002), so it requires nutrients for gamete production potential to be reached (Cicero et al. 2011). Specific requirements of food, mates, and suitable hosts may be needed in order for females to mature additional eggs (Wang and Messing 2003). Subsequent egg maturation is not instantaneous in synovigenic species, and eggs cannot be produced and laid immediately after finding a host (Ellers et al. 2000). Generally, among parasitoid wasps there is little difference in the number of offspring produced between mated and virgin females (King 2002; Riddick 2005), but braconid parasitoids, including *Diachasmimorpha* spp., engage in complex courtship behaviour that may be disrupted by massrearing conditions (Joyce et al. 2010). Knowledge of the effect of mating status on egg load might be useful for estimating production potential of *D. tryoni* in mass-rearing systems.

Although not documented for *D. tryoni*, it is a common finding in parasitoids that large females live longer, have more eggs immediately available for laying, and produce more progeny than their smaller counterparts (Cloutier et al. 2000; Doyon et al. 2005). Research provides strong support that fitness of females increases with size for parasitoid wasps in general (Lauziere et al. 2000). A larger size provides several physiological and behavioural advantages, such as increased lifespan, fecundity, and progeny production (Sagarra et al. 2001).

Studies were conducted to understand the effect of mating status and size on potential fecundity of female *D. tryoni* under laboratory conditions, as well as the effect of 3 carbohydrate sources (honey, white sugar, and golden syrup (56% invert syrup (glucose and fructose), 44% sucrose) at 100mL/L water concentrations) together with oviposition onlifespan and reproductive potential. The experiments addressed (1) whether virgin or mated females produced more offspring and/or matured more eggs, (2) the effect of oviposition on the lifespan of females, (3) the carbohydrate source that best promoted female lifespan and fecundity, and (4) whether female size positively affected egg load and lifespan. The information gained from these experiments will assist in developing massrearing protocols for *D. tryoni* in Australia against the Queensland fruit fly, will have direct relevance to its use in overseas programs against other targets, and will have a bearing on rearing other hymenopteran agents.

## Materials and Methods

### Insect cultures

Cultures of *D. tryoni* and *B. tryoni* were established from infested peaches collected at Gosford Horticulture Institute, Gosford, New South Wales (NSW), Australia, in January 2011. The parasitoid and fruit fly cultures were kept in a growth room (22 ± 2° C, 65 ± 15% RH, 16:8 L:D) at the Elizabeth Macarthur Agricultural Institute (EMAI), Menangle, NSW. Parasitoids were reared on the offspring of field collected *B. tryoni* larvae and supplemented when necessary with *B. tryoni* larvae from the Fruit Fly Production Facility at the EMAI. Adult parasitoids were fed a standard diet of pure honey (streaked on a 50 mL cup using a paintbrush) and water (provided from a dental wick soaked in water) unless used in experiments that specified other treatments. All *B. tryoni* larvae were reared on a standard rehydrated carrot medium diet (Christenson et al. 1956; Snowball et al. 1961) made on site. Adult flies were fed white sugar cubes, yeast hydrolysate (MP Biomedicals, www.mpbio.com), and water.

### Mating status, fecundity, and size

Two treatments were evaluated under laboratory culture conditions: 1) cages with 5 *D. tryoni* females and 5 *D. tryoni* males (mated), and 2) cages with 5 *D. tryoni* females only (virgin). Females were assigned to plastic 175 × 120 × 60 mm cages (model C500, WF Plastic, www.bwfplastic.com/au) upon eclosion and paired with males aged 1 to 10 days. All cages were provided with the standard diet of honey and water and were covered with synthetic gauze mesh secured with 2 rubber bands. Groups of parasitoids (rather than individuals) were kept in the cages so that conditions for mating and oviposition closely resembled conditions experienced during mass rearing. There were 10 cages for the mated treatment (n = 50 females in total) and 7 cages for the virgin treatment (n = 35 females in total); this experiment used parasitoids from 3 generations (F5–7) of *D. tryoni*.

After 10 days in which males and females were allowed to mate and feed, males were removed from the mated cages. Each cage, including the unmated female cages, was then presented with an ovipositional unit on 1 occasion, which comprised a Petri dish containing 28 g of carrot media and approximately 100 third instar *B. tryoni* larvae as hosts (as well as honey and water) for 24 hours. After this time, each Petri dish was removed and placed on a bed of moistened vermiculite (4:1 vermiculite to water) for 10 days, which allowed enough time for all the host larvae present to burrow into the vermiculite and pupate, and then sieved. The number of eclosed parasitoids per treatment was recorded.

To determine the number of progeny per female (progeny yield), the total number of progeny recovered from each ovipositional unit was divided by the number of females that were still alive on the exposure date. After the parent females from both treatments had been exposed to the host larvae, they were killed (frozen) and stored in a -4° C freezer to allow later hind tibia measurements and mature egg load counts (described below).

### Carbohydrate sources, oviposition, and female lifespan

Upon eclosion of F7 parasitoids, *D. tryoni* females were separated and placed individually into plastic cages (as described above). Each female was paired with 1 *D. tryoni* male aged 1 to 10 days (i.e., male:female ratio of 1:1) and provided with 1 of 4 treatments: honey (100 mL/L water), golden syrup (56% invert syrup (glucose and fructose), 44% sucrose) (100 mL/L water), white sugar (cane sugar; 100% sucrose) (100mL/L water), or water only (control). These carbohydrate sources were selected because they are either typically provided in mass-rearing programs (honey, e.g., Wong and Ramadan 1993; Sivinski et al. 1996), are a potentially cheaper form of carbohydrate (sugar) source, or they have been reported in the literature as prolonging the survival and enhancing the reproduction of female parasitoids (maple syrup: primarily sucrose with small amounts of other sugars including fructose and glucose; molasses: sucrose, glucose, and fructose in a ratio of approximately 2:1:1) (e.g., Bautista et al. 2001). In this study, golden syrup was used as a readily available alternative in Australia. There were 10 females for each treatment. When the females were 6 days old, half of the females (5 from each carbohydrate source) were presented with an oviposition unit containing a Petri dish with 28 g of carrot media and approximately 20 third instar *B. tryoni* larvae as hosts. Host material was exposed to the parasitoids for 24 hr, commencing at 10:00 am AEST every 3 days until the females were 12 days old (i.e., days 6, 9, and 12). The remainder of the cages (5 from each carbohydrate source) were not exposed to an ovipositional unit.

**Figure 1.**
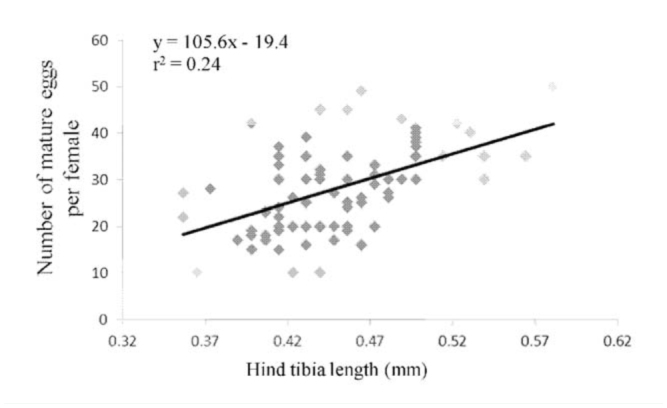
Relationship between adult female *Diachasmimorpha tryoni* size (hind tibia length) and egg load. High quality figures are available online.

Survival of females was recorded daily until death. Females were stored in a -4° C freezer until hind tibia measurements were made.

### Hind tibia and egg load measurements

To determine *D. tryoni* female parasitoid size, hind legs were extended to expose the tibia for measurement with a microscope eyepiece graticule. Females were further analysed for their egg loads under the same microscope settings by removing the abdomen from the rest of the body with tweezers. Small holes were prodded into the abdomen using an entomological pin (0.53 mm diameter). One drop of water was placed over the abdomen to allow for suspension, and then a cover slip was used to squash the abdomen to release the eggs. Eggs were counted as above with a microscope, and numbers recorded.

### Statistical analyses

Separate one-way ANOVAs were used to analyse the effect of mating status on progeny yield and egg load, and the effect of oviposition on lifespan. Linear regression was used to examine the relationship between egg load and female size (hind tibia length). Survival analysis (non-linear regression, Weibull fit) was used to describe the shape of the mortality-time relationship in females from the different carbohydrate treatments. All analyses were conducted using GenStat version 13 (Payne et al. 2010) except the survival analysis, which used JMP (SAS 1995).

## Results

### Mating status, fecundity, and size

Mating status of female *D. tryoni* (10 days old) did not have a significant effect on the mature egg load available for oviposition (*F_1, 83_* = 0.06, *p* = 0.801), with mated females holding an average (± SE) of 28.3 ± 4.0 mature eggs, and virgin females holding an average of 27.8 ± 4.7 mature eggs. The mature egg load of female *D. tryoni* (both mated and virgin) increased with increasing female body size (10.6 eggs for every 0.1 mm increase in hind tibia length; *F_1, 83_* = 26.64, *p* < 0.001; Figure 1).

The mating status of females did not have a significant effect on progeny yield (*F_1, 83_* = 0.58, *p* = 0.448), with mated females producing per female an average of 0.39 ± 0.05 progeny with a 1.2:1 male:female ratio, and virgin females producing per female an average of 0.29 ± 0.05 male progeny.

### Carbohydrate sources, oviposition, and female lifespan

**Figure 2.**
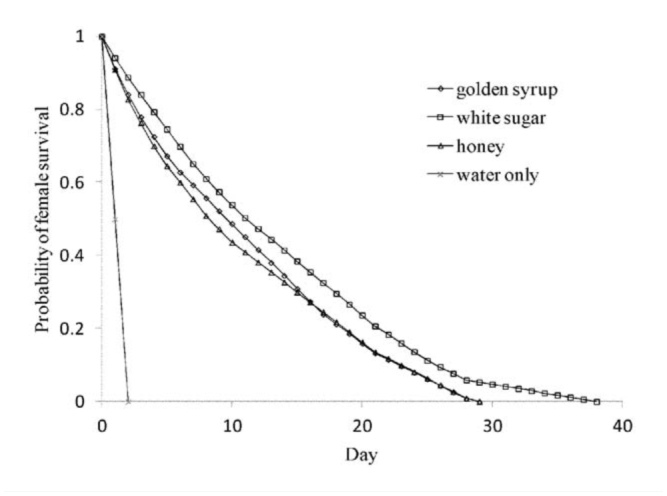
Survivorship curves of *Diachasmimorpha tryoni* fed solutions (100 mL/L water) of golden syrup, white sugar, honey, and water only. High quality figures are available online.

The act of oviposition did not have an effect on female lifespan (*F*_1, 38_ = 0.02, *p* = 0.89). The average (± SE) lifespan of egg laying females was 11.3 ± 2.53, and that of non-egglaying females was 10.8 ± 2.41 days. Data across the oviposition treatments were then pooled to analyse the effect of the carbohydrate treatments on lifespan. Females provided water only, the control, lived for 2.0 ± 0.6 days, those fed honey lived 12.0 ± 3.8 days, those fed golden syrup lived 11.8 ± 3.7 days, and those fed sugar lived 17.9 ± 5.7 days (mean ± SE). Survival analysis across all 4 treatments proved impossible because of the very short lifespan of the parasitoids from the control treatment (Figure 2). Therefore, only the 3 carbohydrate treatments were included in the survival analysis. The shape of the survival curves differed between the 3 carbohydrate sources (c^2^ = 7.90, d.f. = 2, *p* = 0.02, Figure 2) and showed that white sugar gave maximum survival. There was no significant relationship between female size (pooled across the 3 carbohydrate treatments) and lifespan (*F_1, 28_* = 0.03, *p* = 0.87).

## Discussion

The carbohydrate white sugar maximised the survival of female *D. tryoni* under laboratory conditions. Carbohydrates are critical for survival and fecundity of synovigenic parasitoids (Stuhl et al. 2011) such as *D. tryoni*. Another braconid, *Cotesia glomerata* (L.), as well as the ichneumonid *Bathyplectes curculionis* (Thomson), lived longer when fed glucose and other simple monosaccharides compared with more complex carbohydrate solutions (Wäckers 2001; Jacob and Evans 2004). The carbohydrate concentration that maximised the lifespan of 4 braconid species varied from 25–75% (Azzouz et al. 2004; Wu et al. 2008; Lightle et al. 2010; Tompkins et al. 2010), and the relationship between carbohydrate concentration and lifespan was not always linear (Wu et al. 2008). Only a comparatively low concentration (100 mL/L) was used in this study. Higher sugar concentrations are expected to increase lifespan further in *D. tryoni*, but the optimal concentration has not yet been determined. Indeed, if sugar benefits overall performance of the parasitoid (including age-specific fecundity) as effectively as either honey or golden syrup, savings could be made in the cost of materials used in rearing. At $0.21 USD/100 g, sugar is a far cheaper substrate (c.f., honey: $1.16 USD/100 g, and golden syrup: $0.72 USD/100 g).

The act of oviposition did not shorten the lifespan of *D. tryoni*, but the females in this study were only exposed to hosts 3 times during their lifetime, and few progeny emerged. For *Meteorus pulchricornis* (Wesmael) the sugar concentration that maximised lifespan also maximised lifetime progeny production (Wu et al. 2008). Further investigation of the interaction between diet, oviposition, and lifespan is warranted for *D. tryoni* to determine the carbohydrate source that optimises both oviposition and life span, as adult insect feeding allows the utilisation of carbohydrates that may be required for the development of the reproductive system (Jordao et al. 2010). An optimal carbohydrate source such as white sugar will be important to the success of augmentative biological control in conjunction with high quality (large) females (Jacob and Evans 2000; Wäckers 2001).

Female size under laboratory conditions did not have a positive correlation with female lifespan but was positively correlated with egg load for *D. tryoni*. Similarly, the bethylid parasitoid, *Cephalonomia stephanoderis* (Betrem), showed a positive correlation between size and egg load but not lifespan (Lauziere et al. 2000). It is common for larger female parasitoids to live longer (e.g., Sagarra et al. 2001; Doyon and Boivin 2005). The relationships between female size, lifespan, and egg load are strongly influenced by the amount and type of food available and by access to hosts (Godfray 1994), but the nature of these relationships (positive or negative) varies from species to species (Bautista et al. 2001; Eliopoulos et al. 2003; Wang and Messing 2003). Female size accounted for 24% of the variation in mature egg load for *D. tryoni*, indicating that size is only one of the factors to determine egg load in this synovigenic species.

The mating status of *D. tryoni* influenced neither egg maturation nor progeny yield, a proxy measure for attack/parasitism rate. This implies that mating status will not affect the initial efficacy of augmentative biological control, as both virgin and mated females have the capacity to produce similar numbers of mature eggs and therefore progeny. For ongoing biological control it is important that females mate successfully and are able to produce female offspring, but from this study there did not appear to be any advantage of parasitoids mating prior to release. Again, the relationships between mating status, egg maturation, and progeny yield are species-specific, and the effects can be quite subtle (Wang and Messing 2003).

Experiments into improving mass-rearing techniques are vitally important to the success of the augmentative release of biological control agents. The use of white sugar increased female lifespan in the laboratory, which may indicate a need to compare white sugar at varying concentrations with the current practice of providing pure honey. These carbohydrate sources also need to be tested on parasitoids from a wider sampling range, and need to be tested for their effect on other aspects of *D. tryoni* behaviour, such as flight, which is known to be energetically expensive for parasitoids (Hausmann et al. 2005), and foraging in order to gain a better understanding of the carbohydrate source that will optimise the performance of this biological control agent in the field.
